# Middle latency auditory-evoked potential index monitoring of cerebral function to predict functional outcome after emergency craniotomy in patients with brain damage

**DOI:** 10.1186/s13049-015-0161-8

**Published:** 2015-10-20

**Authors:** Junya Tsurukiri, Katsuhiro Nagata, Akira Hoshiai, Taishi Oomura, Hiroyuki Jimbo, Yukio Ikeda

**Affiliations:** Emergency and Critical Care Medicine, Tokyo Medical University Hachioji Medical Center, 1163 Tatemachi, Hachioji, Tokyo, 193-0998 Japan; Neurosurgery, Tokyo Medical University Hachioji Medical Center, 1163 Tatemachi, Hachioji, Tokyo, 193-0998 Japan

**Keywords:** Intensive care unit, Critical care, Emergency medicine, Traumatic brain injury, Stroke, Monitoring

## Abstract

**Background:**

At present, no satisfactory reports on the monitoring of cerebral function to predict functional outcomes after brain damage such as traumatic brain injury (TBI) and stroke. The middle latency auditory-evoked potential index (MLAEPi) monitor (aepEX plus®, Audiomex, UK) is a mobile MLAEP monitor measuring the degree of consciousness that is represented by numerical values. Hence, we hypothesized that MLAEPi predicts neurological outcome after emergency craniotomy among patients with disturbance of consciousness (DOC), which was caused by brain damage.

**Methods:**

The afore-mentioned patients who underwent emergency craniotomy within 12 h of brain damage and were subsequently monitored using MLAEPi were enrolled in this study. DOC was defined as an initial Glasgow Coma Scale score < 8. MLAEPi was measured for 14 days after craniotomy. Neurological outcome was evaluated before discharge using a cerebral performance category (CPC) score and classified into three groups: favorable outcome group for a CPC score of 1 or 2, unfavorable outcome group for a score of 3 or 4, and brain dead (BD) group for a score of 5.

**Results:**

Thirty-two patients were included in this study (17 with TBIs and 15 with acute stroke). Regarding outcome, 10 patients had a favorable outcome, 15 had an unfavorable outcome, and 7 were pronounced BD. MLAEPi was observed to be significantly higher on day 5 than that observed immediately after craniotomy in cases of favorable or unfavorable outcome (63 ± 3.5 vs. 36 ± 2.5 in favorable outcome; 63 ± 3.5 vs. 34 ± 1.8 in unfavorable outcome). MLAEPi was significantly lower in BD patients than in those with a favorable or unfavorable outcome on day 3 (24 ± 4.2 in BD vs. 52 ± 5.2 and 45 ± 2.7 in favorable and unfavorable outcome, respectively) and after day 4. MLAEPi was significantly higher in patients with a favorable outcome than in those with a favorable or unfavorable outcome after day 6 (68 ± 2.3 in favorable outcome vs. 48 ± 2.3 in unfavorable outcome).

**Conclusion:**

We believe that MLAEPi satisfactorily denotes cerebral function and predicts outcomes after emergency craniotomy in patients with DOC, which was caused by acute brain damage.

## Background

Monitoring cerebral function is crucial in surgical critical care. However, to date, there have been no satisfactory reports on the monitoring of cerebral function to predict functional outcome after brain damage, i.e., traumatic brain injury (TBI) and stroke.

Several studies have examined the clinical application of the auditory-evoked potentials (AEPs) as well as the bispectral (BIS) index that provide a good indication of the degree of consciousness under anesthesia in a surgical setting [1]. In particular, cerebral function can be monitored noninvasively by measuring middle latency (ML) AEPs [2]. MLAEPs are derived from AEPs, which reflect the morphology of MLAEP curves, originating from the part of the auditory pathway from the medial geniculate body to the primary auditory cortex. MLAEPs are also less affected by age than other AEP components.

The aepEX plus® (Audiomex, Glasgow, Scotland, UK) is the first mobile and battery-operated MLAEP index (MLAEPi) monitor that can evaluate the level of anesthesia by the numerical value acquired using MLAEP, which is a current generated by excitement of the cranial nerves during information processing in the brain [3]. This monitor is already available worldwide. The aepEX plus® monitoring system is being increasingly used to measure both the level of anesthesia and the cerebral function in intensive care units (ICUs) [1, 4–6]. The recommended range of MLAEPi in general anesthesia settings is 30 to 45. We previously reported the effectiveness of MLAEPi monitoring at the emergency department (ED) in patients with cardiac arrest and disturbance of consciousness (DOC), which was defined as an initial Glasgow Coma Scale (GCS) score < 8, and the recommended range of MLAEPi in non-sedated patients with DOC was between 35 and 61 [7, 8]. In this study, we speculated that MLAEPi predicts neurological outcome after emergency craniotomy among patients with DOC, which was caused by brain damage.

## Materials and methods

### Patients and study design

This prospective study was conducted in the emergency department of Tokyo Medical University Hachioji Medical Center between September 2010 and March 2013. The ethics committees at the institution approved the study design, and written informed consent was obtained from the next of kin of the patients or a posteriori from the patients themselves where possible.

Patients who were admitted to our ED and underwent emergency craniotomy within 12 h of brain damage and were subsequently monitored using MLAEPi were enrolled in this study. DOC was defined as an initial GCS score < 8. We excluded comatose patients younger than 15 years of age, those who had experienced DOC before the onset of brain damage, and those who had drug intoxication, alcoholism, tympanic injury, or terminal diseases during the study.

### AepEX and middle latency auditory-evoked potential index

**Fig. 1:**
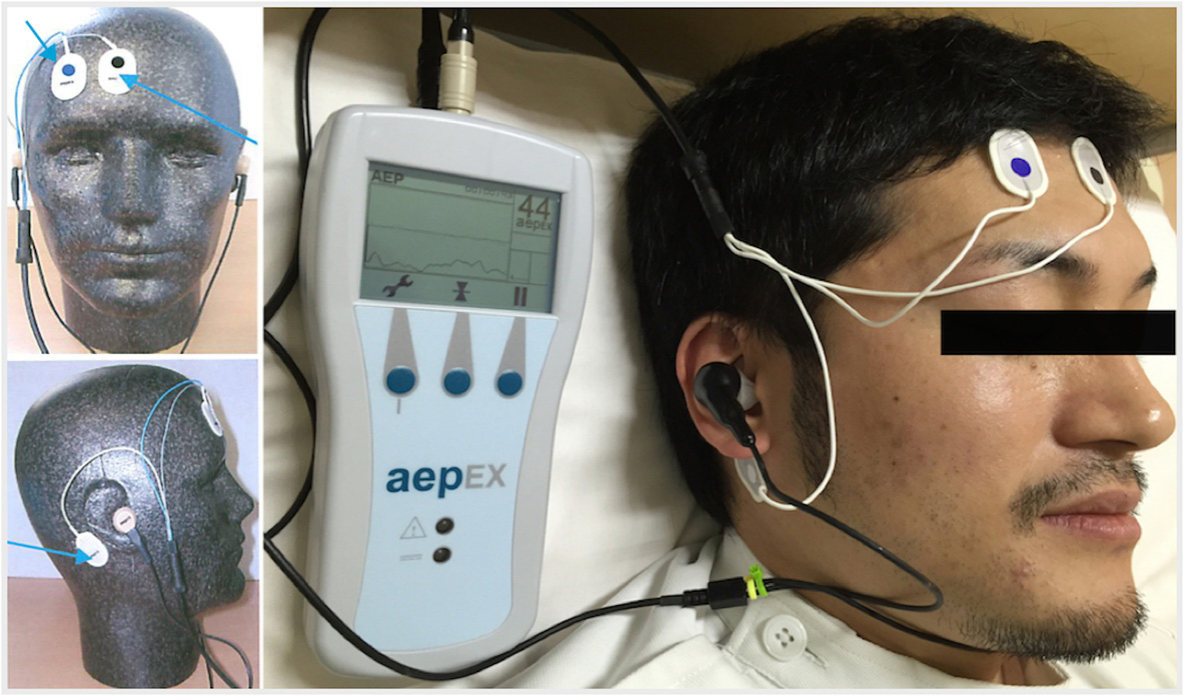
The aepEX plus® monitor

The aepEX plus® monitoring system was activated with bilateral click stimuli through earphones at an intensity < 90 dB for a nominal frequency of 6.9 Hz through a pair of headphones to provide MLAEP. It continuously generates MLAEPi, which is a dimensionless number scaled between 99 (wide awake) and 0 (no brain activity), with differences between successive segments of the curve constructed from its amplitude. The aepEX value is updated every 0.3 s instead of 36.9 s (256 sweeps) by a moving time-average technique. The aepEX plus® identifies the brainstem and cortical components of MLAEP following auditory stimuli, particularly positive Pa and negative Nb waves. MLAEPi is calculated from consistent decreases in amplitude and increases in latency, resulting in individual waves within 144 ms [9]. Detected AEPs were consecutively extracted from the raw electroencephalogram (EEG) signal reflecting the brainstem AEP and MLAEP by an internal processor. The aepEX plus® values were closely related to the AEP waveforms and calculated as the sum of the square root of the absolute difference between every two successive 0.56-ms segments of the AEP waveforms (Fig. 1).

### Intervention

Using the aepEX plus®, MLAEPi was continuously calculated from information provided by disposable sensor EEG electrodes affixed to the patients’ mid (ground electrode) and right (active electrode) forehead as well as the right mastoid (active electrode) after cleaning the skin with 70 % isopropanol. In addition, an emergency medical physician used earphones to determine auditory stimuli. MLAEPi was measured for 14 days after craniotomy every morning and an average of 3 s MLAEPi was recorded for each MLAEPi.

All patients were administered sedatives for no longer than 3 days after the onset of brain damage. All procedural sedations were performed by expert emergency physicians, and the choice of sedatives was at the discretion of the emergency physician. In our ICUs, sedation was achieved using midazolam (maximum 0.2 mg/kg/h) or propofol (maximum 3 mg/kg/h) as a sedative and fentanyl (maximum 1.5 g/kg/h) for pain management. Neurological outcome was evaluated before discharge using a cerebral performance category (CPC) score 1 month after brain damage and classified into the following three groups: favorable outcome group for a CPC score of 1 or 2; unfavorable outcome group for a score of 3 or 4; and brain dead (BD) group, which equated to a score of 5 (Table 1).

**Table 1:** Cerebral performance category

Favorable outcome	
CPC 1	Mild deficits. Able to work. May have mild neurologic or psychologic deficits
CPC 2	Moderate deficits. Capable of independent activities of daily life. Able work in sheltered environment.
Unfavorable outcome	
CPC 3	Severe deficits. Conscious but dependent on others for daily support. Ranges from ambulatory state to severe dementia or paralysis.
CPC 4	Coma or vegetative state
Brain dead	
CPC 5	Apnea, areflexia, EEG silence

### Data collection

The following characteristics were noted from the charts of the comatose patients with brain damage: age, gender, initial GCS, vital signs, clinical history, value of MLAEPi, and etiologies of brain damage. Continuous MLAEPi monitoring did not affect standard intensive care treatment and nursing in the ICU.

### Statistical analyses

Data from all eligible patients were analyzed. Continuous variables were shown as median values with interquartile ranges. Intergroup differences were statistically assessed using the Kruskal–Wallis test and one-way ANOVA with repeated measures, depending on the distribution of measured variables using Prism version 6.0a statistical software (GraphPad Software, San Diego, CA, USA).

## Results

### Clinical characteristics

Thirty-two comatose patients with brain damage (median age was 55 years) were included in this study. The demographics and clinical characteristics of patients are shown in Table 2. The GCS score of E1V1M2 was common in this study. In the cohort, 17 patients had TBI and 15 had acute stroke. The etiologies of brain damage of patients are shown in Table 3. Regarding outcome, 10 patients had neurologically favorable outcome, 15 had unfavorable outcome, and 7 were pronounced BD.

**Table 2:** The demographics and clinical characteristics of patients

Clinical characteristics	
Variables	*n* = 32
Age (y), medium (IQR)	55 (44–71)
Male, n (%)	18 (56)
Glagow Coma Scale score, *n* (%)	
3	3 (9)
4	11 (33)
5	1 (3)
6	6 (18)
7	3 (9)
8	8 (25)
Etiologies of brain damage, *n* (%)	
Traumatic brain injury	17 (53)
Intraranial hematoma	5 (15)
Subarachnoid hemorrhage	8 (24)
Cerebral infarction	2 (6)
Neurological outcomes, *n* (%)	
Favorable	10 (31)
Unfavorable	15 (47)
Brain death	7 (21)

*IQR* interquartile range

**Table 3:** Etiologies of brain damage of patients

Main brain damage	
Trauma	*n* = 17
Subdural hematoma, *n* (%)	
Left	7 (41)
Right	7 (41)
Bilateral	1 (6)
posterior cranial fossa	1 (6)
Epidural hematoma, *n* (%)	
Left	2 (12)
Right	3 (18)
Cerebral contusion, *n* (%)	15 (88)
Diffuse brain injury, *n* (%)	1 (6)
Intracranial hematoma, *n* (%)	*n* = 8
Putamen	1 (13)
Thalamus	2 (25)
Subcortex	2 (25)
Arteriovenous malformation	3 (28)
Subarachnoid hemorrhage, n (%)	*n* = 5
Ruptured aneurysm (Fisher group 3)	5 (100)
Cerebral infaction, *n* (%)	*n* = 2
Cerebellum	2 (100)

### Changes in MLAEPi at ICU

**Fig. 2:**
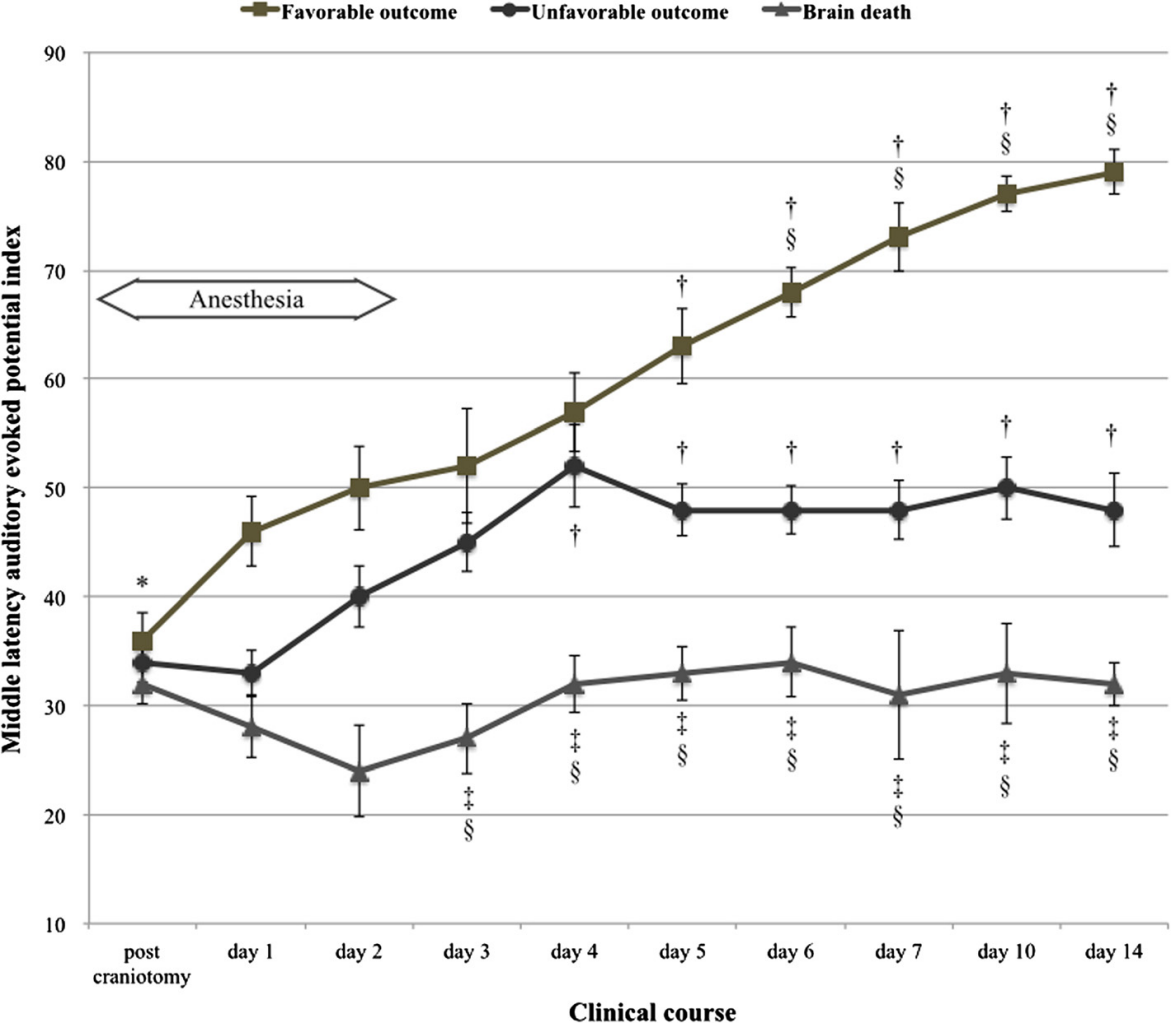
Changes in MLAEPi value with a standard error at the intensive care unit. * = non-significance; † = *p* < 0.01 vs. post-craniotomy; ‡ = *p* < 0.01 vs. favorable outcome group; § = *p* < 0.01 vs. unfavorable outcome group

MLAEPi values measured until 14 days after injury are shown in Table 4. During patient sedation for 3 days after injury, MLAEPi did not significantly differ among the favorable outcome, unfavorable outcome, and BD groups. However, MLAEPi was observed to be significantly higher on day 5 than that observed immediately after craniotomy in the favorable outcome (*p* < 0.01) and unfavorable outcome groups (*p* < 0.01). MLAEPi was also observed to be significantly lower in the BD group than that in the favorable outcome and unfavorable outcome groups after day 3 (*p* < 0.01). Furthermore, MLAEPi was significantly higher in the favorable outcome group compared with that in the unfavorable outcome group after day 6 (*p* < 0.01). MLAEPi in the BD group did not show any significant increase during study periods (Fig. 2).

**Table 4:** Measures of middle latency auditory-evoked potential index

Median MLAEPi value with a standard error			
	Favorable outcome	Unfavorable outcome	Brain death
	(*n* = 10)	(*n* = 15)	(*n* = 7)
Post craniotomy	36 ± 2.5	34 ± 1.8	32 ± 1.8
Day 1	46 ± 3.2	33 ± 2.1	28 ± 2.8
Day 2	50 ± 3.8	40 ± 2.8	24 ± 4.2
Day 3	52 ± 5.2	45 ± 2.7	27 ± 3.2
Day 4	56 ± 3.6	52 ± 3.7	32 ± 2.6
Day 5	63 ± 3.5	48 ± 2.4	33 ± 2.5
Day 6	68 ± 2.3	48 ± 2.3	34 ± 3.2
Day 7	73 ± 3.1	48 ± 2.7	31 ± 5.9
Day 10	77 ± 1.6	50 ± 2.9	33 ± 4.6
Day 14	79 ± 2.1	48 ± 3.4	32 ± 2.0

*MLAEPi* middle latency auditory evoked potentials index

## Discussion

To our knowledge, this is the first prospective study to evaluate the changes of MLAEPi in patients with brain damage in an ICU. Several studies have found that white matter tracts within the cerebral hemisphere and brain stem are injured following TBI or acute stroke [10, 11]. MLAEP is usually obtained intermittently and derived from AEPs, which reflects the morphology of MLAEP curves. Previous studies have failed to demonstrate the usefulness of single components of the early somatosensory-evoked potentials (SEP) and short latency AEP for predicting the clinical outcome after TBI or acute ischemic injuries following stroke or cardiac arrest [12, 13]. Although middle latency activities are considered to be good predictors of prognosis in comatose patients, the predictive value of MLAEP remains uncertain. Therefore, we hypothesized that MLAEPi decreases among patients with DOC caused by head injury or acute stroke and can predict the neurological outcomes after emergency craniotomy.

Most studies have evaluated MLAEPi as an indicator of the state of anesthesia with 100 % specificity using an MLAEPi cut-off value of 37 for unconsciousness during anesthesia [14]. We previously demonstrated that the recommended range of MLAEPi in non-sedated patients with DOC between 35 and 61 [8]. MLAEPi is profoundly affected by the decreasing amplitudes and increasing latencies induced by hypnotic drugs such as midazolam and propofol. Although the recommended range of MLAEPi in surgical anesthesia settings is 30 to 45, the adequate range of MLAEPi for critical care patients in ICUs remains unknown [3]. Our results show that MLAEPi did not differ significantly in patients with brain damage across the three outcome groups during sedative periods. However, we were able to demonstrate that MLAEPi increased significantly between patients with favorable outcome or unfavorable outcome compared with patients with BD immediately after finishing sedation. Moreover, MLAEPi increased significantly among patients with favorable outcome compared with patients with unfavorable outcome within 1 week after the onset of brain damage.

Several studies found that the GCS score and BIS value were relatively correlated in critically ill patients, and the processed EEG is a non-invasive method for monitoring consciousness during anesthesia or critical care sedation [9, 15]. BIS is the numerical value acquired using spontaneous EEG. Although a BIS value of 0 is useful in severe TBI or ischemic brain injury for the early detection and confirmation of BD with a GCS score of 3, there has been no satisfactory report on BIS monitoring of cerebral function to predict functional outcome after brain damage [16, 17]. Middle latency SEP may be valuable for increasing sensitivity without any loss of specificity for predicting unfavorable outcome in patients after stroke, ischemic brain injury or TBI [13, 18]. However, the prognostic value of SEPs remains controversial and should never be considered in isolation but should be integrated with other neurophysiological tools and clinical examination [19]. It has been reported that aepEX monitoring is a more effective indicator for determining the state of consciousness than BIS or any other EEG-based monitoring method [4, 5, 14, 20]. The major difference between any other EEG-based monitoring method and the aepEX method is that MLAEPi is the numerical value acquired using MLAEP. The strong correlation between GCS scores and MLAEPi in patients with DOC has been previously reported [7, 8]. Furthermore, because of its small size and battery operation facility, aepEX monitoring can provide a consistent assessment of MLAEP during life-saving procedures while transporting patients within the hospital and in patients admitted to ICUs. In this study, we demonstrated that MLAEPi may be a reasonable indicator of neurological outcomes in patients with brain damage who have underwent emergency craniotomy in an acute care setting.

This study has several limitations, particularly the small number of evaluated patients sustaining different types of brain damage such as focal TBI, diffuse TBI, and cerebral stroke. Second, we did not measure initial MLAEPi in ED and did not use other monitors such as BIS, short latency AEP, early SEP, or MLAEP to evaluate the degree of DOC in the ICU. Third, the measurement of MLAEPi was performed by a single emergency physician (Dr. TJ). Thus, the patients with DOC were not enrolled sequentially in this study. Fourth, we only obtained MLAEPi data for a period of up to two weeks and had no records from the late phase following discharge from the hospital. The purpose of this study was to assess MLAEPi monitoring for predicting functional outcomes in the acute phase in patients admitted to ICUs. Thus, we limited the study endpoint to the initial evaluation in the acute phase and did not perform long-term MLAEPi follow-up.

## Conclusion

MLAEPi can satisfactorily denote cerebral function as represented by simple numerical values, and predict functional outcomes after emergency craniotomy among patients with DOC, which was caused by brain damage. Large studies are essential for further evaluating the reliable cut-offs of MLAEPi in patients with acute brain damage.

## Abbreviations

AEP: Auditory-evoked potential
BD: Brain dead
BIS: Bispectral index
CPC: Cerebral performance category
DOC: Disturbance of consciousness
ED: Emergency department
EEG: Electroencephalogram
GCS: GLASGOW Coma Scale
ICU: Intensive care unit
MLAEP: Middle latency auditory-evoked potential
SEP: Somatosensory-evoked potentiala
TBI: Traumatic brain injury
